# Adherence to eHealth Interventions Among Patients With Heart Failure: Scoping Review

**DOI:** 10.2196/63409

**Published:** 2025-06-27

**Authors:** Arno Joachim Gingele, Bianca Steiner, Bettina Zippel-Schultz, Hans-Peter Brunner-La Rocca

**Affiliations:** 1 Department of Cardiology, Maastricht University Medical Center, P Debyelaan 25, 6229 HX, Maastricht, The Netherlands, 31 043 387 6543; 2 German Foundation for the Chronically Ill, Berlin, Germany

**Keywords:** heart failure, eHealth, patient adherence, review, quantitative data, mixed model, wearables, invasive devices, telephone support

## Abstract

**Background:**

Heart failure (HF) is a significant global health challenge, requiring innovative management strategies like eHealth. However, the success of eHealth in managing HF heavily relies on patient adherence, an area currently not sufficiently investigated despite its critical role in ensuring the effectiveness of this approach.

**Objective:**

This review was initiated to gather evidence on adherence to eHealth devices among patients with HF. The goal was to survey the current state of adherence, pinpoint factors that promote successful engagement, and identify gaps needing further research.

**Methods:**

A scoping review was conducted to gather quantitative data on eHealth engagement from relevant clinical HF studies indexed in PubMed, CINAHL, and PsycINFO up to February 2025. Descriptive characteristics of the publications were extracted, and generalized mixed model analyses were used to identify eHealth characteristics affecting patient adherence.

**Results:**

Our analysis included 70 studies, primarily using noninvasive eHealth interventions with wearables (n=51), followed by wearables only (n=8), noninvasive eHealth interventions without wearables (n=6), invasive devices (n=3), and telephone support (n=2). The median number of patients per study was 49 (IQR 20‐139), and the median follow-up duration was 180 (IQR 84‐360) days. Variability in reporting and definitions of eHealth adherence was noted. In total, 20 studies assessed adherence trends, with 13 noting a decline, 6 observing no change, and 1 reporting an increase over time. Factors influencing adherence were explored in 29 studies; 7 indicated higher adherence with increasing patient age, 2 showed a negative correlation, and 9 detected no age-related differences. No gender differences were found in the 10 publications that reported on gender, and 9 studies found no association between adherence and the New York Heart Association classification, while 1 noted higher adherence in patients with more severe symptoms. In 35 (50%) studies, adherence was quantified as the percentage of mean days the intervention was used, yielding a median adherence rate of 78% (IQR 61%‐86%; range 31%‐98%). No significant correlations were found between adherence rates and the number of eHealth device users, type of intervention, follow-up duration, number of parameters monitored, or data collection frequency.

**Conclusions:**

Reporting and definitions of patient adherence in HF studies are incomplete and inconsistent. Trends indicate a decrease in eHealth use over time. Customizing devices to meet patient needs may help mitigate this issue. Future research should offer a more detailed description of adherence to pinpoint factors that enhance patient adherence with eHealth technologies.

## Introduction

### Background

Heart failure (HF) is a critical global health issue marked by high morbidity and mortality, challenging health care systems with its complex management needs and significant resource use [1,2]. The aging population is expected to drive a 46% increase in HF prevalence over the next decade [3], necessitating innovative management strategies to address the growing burden [4].

eHealth, defined as the use of information and communication technologies to support health services or information, offers the potential to revolutionize HF care by enhancing access to health care services, improving patient outcomes, and reducing the necessity for hospital readmissions [5]. Evidence suggests that eHealth interventions like web-based remote patient management systems or mobile apps can significantly improve morbidity and mortality outcomes in patients with HF, presenting a viable pathway to mitigate the burden of this condition [6]. For instance, the Optimization of the Ambulatory Monitoring for Patients With Heart Failure by Tele-cardiology trial demonstrated that telemonitoring reduced HF hospitalizations in patients with more advanced HF [7].

However, the effectiveness and potential benefits of eHealth in HF management are contingent upon patient adherence to the technologies used [8]. Adherence in eHealth has been defined by Donkin et al [9] as “the degree to which the user followed the program as it was designed” and is influenced by various technological (eg, front-end design) and individual (eg, digital literacy) factors [10]. Despite the acknowledged importance of adherence, there is a paucity of research exploring this aspect, particularly in the context of the diverse range of eHealth devices available, each with its own set of functionalities [6]. Existing reviews on this topic are scarce and often focus on specific subdomains of eHealth, such as mobile health (mHealth) [11], or examine adherence in chronic diseases more broadly rather than addressing HF specifically [12]. Understanding the factors that influence adherence to eHealth in HF is essential for the development and implementation of effective interventions [13].

Consequently, our study embarked on a scoping review to collate evidence on adherence to eHealth devices within the patient population with HF. The aim was to provide an overview of the current state of adherence, identify the determinants of successful engagement, and highlight areas requiring further investigation. This endeavor is critical for informing future strategies to enhance the utility and impact of eHealth solutions in managing HF, ultimately contributing to better patient outcomes and more sustainable health care systems.

### Objectives

The primary objective of our literature analysis was to present an overview of eHealth adherence in HF studies, describing trends in adherence and identifying patient characteristics associated with improved adherence. The secondary objective was to identify eHealth characteristics linked to better adherence by assessing the relationship between adherence and these characteristics. Such insights could inform the design of future eHealth interventions by incorporating characteristics that promote sustained adherence.

## Methods

### Overview

The scoping review conducted to synthesize evidence regarding engagement with eHealth followed the guidelines outlined in the PRISMA-ScR (Preferred Reporting Items for Systematic Reviews and Meta-Analyses extension for Scoping Reviews) checklist, as detailed in Checklist 1 [14].

### Information Sources

The search for clinical studies was conducted through the PubMed, CINAHL, and PsycINFO databases, encompassing literature published up to February 2025.

### Search Strategy

The keywords used in our search were “heart failure,” “adherence,” and “eHealth,” combined with the MeSH (Medical Subject Headings) terms “Heart Failure,” “Patient Compliance,” and “Telemedicine” (see Multimedia Appendix 1 for the exact searching strategy, including search terms used for PubMed, PsycINFO, and CINAHL). Our search strategy was guided by the population, intervention, comparison, and outcome framework.

- Population: Studies involving patients with HF were included due to the significant impact of HF on health care systems.
- Intervention: Studies that involved eHealth interventions were included, as they have the potential to improve HF care by providing more accessible and continuous care.
- Comparison: Clinical studies, with or without a comparison group, were included. This broad inclusion criterion ensured that a wide range of evidence was captured.
- Outcome: Studies reporting on patient adherence were included, as improved adherence is crucial for the success of eHealth interventions.

### Study Selection

Studies qualified for inclusion if they were peer-reviewed and investigated the impact of eHealth interventions on patients aged 18 years or older diagnosed with HF across its entire spectrum (HF with reduced ejection fraction, HF with mildly reduced ejection fraction, or HF with preserved ejection fraction). Additionally, the publications needed to report quantitative system use data to enable objective measurement of patient adherence to the eHealth device. The paper had to be written in English. Study protocols, systematic reviews, qualitative studies, meta-analyses, and conference abstracts were excluded.

### Data Collection

Sourced studies were assessed for full-text eligibility by a dual independent review performed by AJG and HPB-LR. In case of disagreement, a third reviewer (BZ-S) was consulted for the final decision. Data extraction was executed by AJG using a structured extraction form to ensure consistency and completeness. For each study, the following study characteristics were extracted: title of the study, name of the first author, year of publication, number of patients included in the eHealth intervention group, type of eHealth intervention (noninvasive eHealth without wearables, noninvasive eHealth with wearables, invasive eHealth, telephone support, and wearables only), description of the eHealth intervention implemented, duration of intervention, overview of adherence metrics, and any factors associated with adherence as reported by the authors. To enhance accuracy, the extracted data were cross-checked for completeness and correctness, and any uncertainties were resolved through discussion with the coauthors.

### Statistical Analysis

Descriptive statistics were used to describe the study characteristics of included studies, trends in adherence, and patient characteristics associated with adherence. To assess the relationship between adherence and eHealth characteristics (type of eHealth intervention, duration of intervention, number of patients with eHealth, number of parameters collected by eHealth, and frequency of data collection), a generalized mixed model analysis was performed. As it was expected that definitions and reporting of adherence might vary significantly between studies, adherence was defined for this analysis as the mean percentage of days the intervention was used. Studies that did not report adherence in this manner or did not provide sufficient data to calculate it were excluded from the inferential statistics. A *P* value of .05 or less was considered to be statistically significant. All analyses were performed in SPSS (version 28; IBM Corp).

## Results

### Description of Study Characteristics

**Figure 1.**
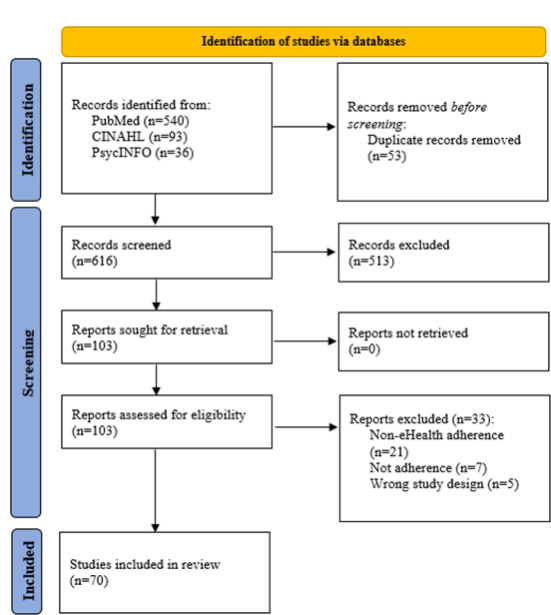
PRISMA (Preferred Reporting Items for Systematic Reviews and Meta-Analyses) flowchart for study selection.

A total of 669 titles were initially identified (Figure 1). Subsequently, title and abstract screening was conducted, resulting in 103 records being deemed potentially relevant. Following a detailed assessment of the full texts for eligibility, 70 papers were ultimately included in the review.

Among the 70 studies included, the majority used noninvasive eHealth devices with wearables (n=51), followed by wearables only (n=8), noninvasive eHealth devices without wearables (n=6), invasive eHealth devices (n=3), and telephone support (n=2). Among the studies reporting on wearables, the most commonly used devices were weight scales (n=44), blood pressure monitors (n=32), activity trackers (n=11), and electrocardiogram recorders (n=8). The most frequent combination was a weight scale and a blood pressure monitor (n=31). Median number of patients per study using an eHealth device was 49 (IQR 20‐139, range 9‐3449). Median duration of follow-up was 180 (IQR 84‐360, range 7‐1023) days.

### Description of eHealth Adherence Across Studies

The reporting of eHealth adherence exhibited substantial variation across studies, including differences in the definitions used. For example, Koehler et al [15] reported adherence as the number of patients with at least 70% of daily data transfers and no break for >30 days (except during hospitalizations), whereas Kastner et al [16] described adherence as a number of days a complete set of patient data was sent. Additionally, adherence documentation was frequently concise and lacked detail. The diversity of devices used and the types of data collected (eg, vital parameters and symptoms) complicated the pooling of results (see Multimedia Appendix 2 [7,15-83] for detailed study characteristics of all included studies).

In total, 20 (29%) studies explored trends in adherence: 13 reported a decline in adherence over the course of the follow-up, 6 found no change, and 1 study noted improved adherence throughout the study period. For example, Ware et al [17] reported a decline in adherence to a noninvasive eHealth intervention with wearables, defined as the percentage of days all patient data were transmitted, from 81.2% in the first month to 63.1% after 12 months.

### Association Between Adherence and Patient Characteristics

In 29 (41%) studies, factors influencing eHealth adherence were reported (Table 1). Among these, 7 studies identified a positive association between patient age and adherence, indicating that older patients were more likely to adhere to eHealth device protocols. Conversely, 2 studies reported a negative association between patient age and adherence levels, while 9 studies observed no association. Regarding the impact of gender on adherence, all 10 studies examining this variable found no significant association between gender and eHealth adherence. Furthermore, 9 of 10 studies reported no association between the New York Heart Association (NYHA) functional classification and eHealth adherence, and 1 study found increased adherence among patients exhibiting more severe symptoms. Besides age, NYHA classification, and gender, most studies reported on living status (n=7), educational level (n=5), and race (n=4). Additionally, 31 factors were mentioned once, including loss of interest and mental overload.

**Table 1.** Factors associated with adherence*.*

Author (year of publication)	Study characteristics	Intervention	Adherence	Factors reported (direction of association, level of significance)
Yoon et al (2024) [18]	n^a^=39 Country: Korea Duration: 1 month	App with Bluetooth-enabled wearables	App: 80% (mean number of days with logins)	Age (−^b^, s^c^)
Kagiyama et al (2024)[19]	n=77 Country: Japan Duration: 6 months (median)	Digital tablet with Bluetooth-enabled wearables	Overall: 75% (patients with ≥70% of days with self-measurement)	Age (+^d^, s) QoL^e^ measured with MLHFQ^f^ (−, s) *Gender^g^, BMI, residence type, past HF^h^ hospitalization, NYHA^i^, QoL measured with EQ-5D^j^*
Brons et al (2023)[20]	n=150 Country: The Netherlands Duration: 12 months	e-Vita HF web-based platform with wearables	Weight: 74% (patients with 3 or more data transmissions a week for at least 42 weeks within 1 year) Weight, blood pressure, and heart rate combined: 67%	HF hospitalizations (−, s) *Age, gender, education level, living alone, BMI, smoking, comorbidities, NYHA, LVEF^k^, duration of HF, self-care*
Prescher et al (2023)[21]	n=670 Country: Germany Duration: 12 months	Digital tablet with Bluetooth-enabled wearables	Overall: 89.1% (days all parameters collected) Blood pressure monitor: 93.71%; weight scale: 92.4% Electrocardiogram: 92.1%	Age (+, s) Cardiovascular hospitalization (−, s) Rural areas (+, s) ICD^l^ (−, s) *Gender, DM^m^, NT-proBNP^n^, living alone, CRT^o^, LVEF, NYHA*
Ziacchi et al (2023)[22]	n=138 Country: Italy Duration: 12 months	MyTriage app	Overall: 49.3% (mean)	Age (+, s) NYHA (+, s) School qualification (−, s) Assistance by caregiver in app use (+,s)
Barbaric et al (2022)[23]	n=20 Country: Canada Duration: 1 month	Medly voice app	Overall: 73%	Age (+) Confidence in using technology (+)
Sabatier (2022)[24]	n=659 Country: France Duration: 4 months	Web-based telehealth platform	Overall: 65.7% (mean of anticipated monitoring sessions)	Visual analog scale moral score (+, s) *Comorbidities, NYHA, LVEF, treatment adherence*
Apergi et al (2021)[25]	n=47 Country: United States Duration: 3 months	Amazon’s Alexa+ or Avatar (via tablet)	Alexa+: mean use of 35.3 times Avatar: mean use of 37.8 times	Age (+, s) Number of HF medications (−, s) *Race, household income, or confidence in using technology*
Ploux et al (2021)[26]	n=51 Country: France Duration: 2 months	CareLine Solutions app with wearables	Before lockdown: 84% During lockdown: 87%	Age (+) Confidence in using technology (+)
Guzman-Clark et al (2021)[27]	n=3449 Country: United States Duration: 12 months	Telehealth website with wearables	Overall: 57.1% (mean number of days patients logged in)	Age (−, s) White race (−, s) Severity of illness (−, s)
Haynes et al (2021)[28]	n=292 Country: United States Duration: 6 months	Monitor with Bluetooth-enabled wearables	Overall: 49% (days weight was transmitted)	*Emergency department visits*
Zisis et al (2021)[29]	n=10 Country: Australia Duration: 2 months	HF app (via tablet)	Overall: 20% of participants completed ≥70% of the full program, 80% did not engage at all	*Age, education level, QoL, HF knowledge, self-care, anxiety, cognition*
Radhakrishnan et al (2021)[30]	n=15 Country: United States Duration: 3 months	Heart Health Mountain app with digital gaming	Weight: 80% (patients transmitted weight data for 5 or more days a week)	Use of digital gaming (+, s)
Sohn et al (2020)[31]	n=20 Country: United States Duration: 6 months	FitBit activity tracker with BodyTrace scale and smart pill bottles	FitBit: 79.1% (median number of hours activity tracker was worn) Bathroom scale: 59.7% (days scale was used) Smart pill bottle: 2.8% (days bottle was used)	SCHFI^p^ confidence subscale scores (−, s) (FitBit) SAQ^q^ scores (−, s) (FitBit) SCHFI confidence subscale scores (−, s) (Body scale) *Gender, age, race, NYHA, ejection fraction, education, annual income*
Ding et al (2020)[32]	n=91 Country: Australia Duration: 6 months	Bluetooth-enabled weight scale	Weight: 74% (patients with 4 or more days with data transmission a week)	Being away from home (−) Technical issues (−) Hospitalizations and emergency department presentations (−) Being unwell, falling, surgery, and chemotherapy (−)
Haynes et al (2020)[33]	n=538 Country: United States Duration: 6 months	Telehealth device with Bluetooth-enabled wearables	Weight: 53.3% (study days with weight transmission)	Weekend (−) Winter months (−)
Haynes et al (2020)[34]	n=12 Country: United States Duration: 34 months (median)	CardioMEMS device (sensor in the pulmonary artery), CardioMEMS pillow	Overall: 77.6%	Participants self-identified as committed, capable, eager to follow instructions, and generally do things well (+)
Hovland-Tånneryd et al (2019) [35]	n=82 Country: Sweden Duration: 6 months	OPTILOGG device (app with wearables)	Overall: 94% (median number of days patients used the eHealth tool)	Living in rural areas (+) *Age, gender*
Ware et al (2019)[17]	n=232 Country: Canada Duration: 12 months	Medly smartphone app with wearables	Overall: 73.6% (days patients took all readings)	Time device is used (−) Age (+) *NYHA, gender*
Rosen et al (2017)[36]	n=48 Country: United States Duration: 6 months	Telehealth platform (via tablet) with Bluetooth-enabled wearables	Overall: 96% (median number of days, data were transmitted)	*Gender, race, age, living situation, depression, cognitive ability, risk for readmission*
Siebermair et al (2015)[37]	n=159 Country: Germany Duration: 21 months (mean)	CareLink system (handheld telemetry wand for data transmission of implanted devices)	Initial data transmission: 76.1% (patients completing transmission)	Loss of interest (−) Mental overload (−) Lack of support by the responsible general practitioner (−) Concerns about privacy of relevant health-related device data (−) Doubts regarding the telemonitoring concept (−) Technical problems (−)
Prescher et al (2014)[38]	n=354 Country: Germany Duration: 26 months (median)	App with wearables	Overall: 88.9% (patients transmitting at least 1 vital parameter a day)	*Age, gender, NYHA, LVEF, depression, diabetes, NT-proBNP, BMI, living alone, duration of HF*
Guzman-Clark et al (2013)[39]	n=248 Country: United States Duration: 3 months	Health Buddy (portable telemonitoring device)	Overall: 54.9 days (mean number of days with response per study days)	Primary care (+) Comorbidity burden (−, s) *Age, marital status, gender, caregiver available, income, insurance*
Seto et al (2012)[84]	n=50 Country: Canada Duration: 6 months	App with Bluetooth-enabled wearables	Overall: 70% (patients completing at least 80% of their possible daily readings)	Planned absence (+) Equipment failure (+)
Morak et al (2011)[40]	n=21 Country: Austria Duration: 1 week	App with Near Field Communication–enabled wearables	Overall: 82.2% (at an expected rate of 13 datasets per patient)	Technical problems (−)
Mortara et al (2009)[41]	n=301 Country: Italy Duration: 12 months	Telephone support with wearables	Vital signs: 81% (requested data that were transmitted); cardiorespiratory recordings: 92%	*NYHA, age*
Piette et al (2008)[42]	n=52 Country: United States Duration: 3 months (mean)	Touch-tone telephone support	Overall: 92% (successful assessment attempts)	*Age, severity of HF, education*
Clark et al (2007)[43]	n=79 Country: Australia Duration: 12 months	Telephone support	Overall: 65.8% (patients submitting data once a month or more)	*Gender, age, living status, weight, NYHA, rural area, comorbidities, HF medication*
de Lusignan et al (2001)[44]	n=10 Country: United Kingdom Duration: 12 months (mean)	Video consultation equipment with wearables	Weight: 74% (mean number of days data were transmitted) Blood pressure and pulse watch: 90%	Planned absence and equipment failure (−) (blood pressure and pulse watch)

a n=number of patients with eHealth intervention.

b “−” indicates negative association with adherence.

c s=association statistically significant.

d “+” indicates positive association with adherence.

e QoL: quality of life.

f MLHFQ: Minnesota Living with Heart Failure Questionnaire.

g Factors in italics format indicate no association with adherence.

h HF: heart failure.

i NYHA: New York Heart Association.

j EQ-5D: EuroQol 5 dimensions questionnaire.

k LVEF: left ventricular ejection fraction.

l ICD: implantable cardioverter defibrillator.

m DM: diabetes mellitus.

n NT-proBNP: N-terminal pro-brain natriuretic peptide.

o CRT: cardiac resynchronization therapy.

p SCHFI: Self-Care of Heart Failure Index.

q SAQ: Seattle Angina Questionnaire.

### Association Between Adherence and eHealth Characteristics

In 35 (50%) studies, adherence was either reported directly or could be inferred as the mean percentage of days the intervention was used (see Multimedia Appendix 2 for detailed study characteristics of all included studies). For these studies, median adherence was 78% (IQR 61%‐86%; range 31%‐98%). No significant correlations were found between adherence rates and predefined eHealth characteristics (Table 2). Telephone support was not detailed in the included studies, and therefore, was not incorporated into the analysis. In the other 35 (50%) studies, adherence was reported in a different manner. For instance, these studies might report the number of patients using the application or solely providing a percentage without further definitions. Therefore, they were excluded from this analysis.

**Table 2.** Correlation of eHealth characteristics and adherence*.*

eHealth characteristics	Wald chi-square (*df*=1)	*P* value
Frequency of data collection	1.057	.30
Number of patients with eHealth	0.070	.79
Duration of intervention	0.002	.97
Number of parameters collected by eHealth	0.306	.58
Type of eHealth intervention (reference category: noninvasive eHealth with wearables)		
Noninvasive eHealth without wearables	0.332	.57
Invasive eHealth	0.053	.82
Wearables only	0.376	.54

## Discussion

### Principal Findings

The reporting of adherence of patients with HF with eHealth interventions across the included studies was limited and lacked uniformity, which hindered the identification of influencing factors. Despite this, adherence to eHealth interventions was generally high, though there was a large variation between studies. In addition, it tended to decrease over time.

As HF is a chronic disease, its treatment requires lifelong therapy. We observed a trend toward decreased eHealth use over time, a finding corroborated by Nelson et al [85], who documented a similar decline in eHealth use among patients with diabetes mellitus. This pattern is also evident in pharmacological HF therapy, where patient adherence decreases over time [86]. A potential explanation is that patient interest may wane, as the duration of an intervention extends. This issue deserves more attention in general, as our results indicated that loss of interest was reported as a factor influencing eHealth adherence in only one of the included studies. To bolster long-term adherence, it might be crucial to tailor eHealth devices to meet the specific needs of patients [87]. The information provided by the device should be suitable for the patient’s cognitive abilities and cultural background. Additionally, the design and user interface should align with the patient’s digital experience and interests. As patients will be using the device for years, updating patient education and self-care information in various formats could enhance patient engagement as well. Moreover, adherence to self-care behaviors might vary depending on the specific task. Physical activity is likely the most feasible to track, as it can be measured passively using a smartwatch or activity tracker, whereas behaviors such as weight monitoring or symptom reporting require active patient input, potentially leading to lower adherence. As evidence suggests that changes in functional status can serve as an early indicator of impending decompensation [88], integrating this parameter more routinely into HF care could enhance disease management.

A significant obstacle to the implementation of eHealth solutions in health care is the concern that patients may be overwhelmed by the additional workload imposed by the devices, potentially leading to the absence or discontinuation of use [89]. Still, no clear trends indicating lower adherence for devices requiring more interaction (such as those monitoring more parameters or collecting data more frequently) were observed in our study. Moreover, none of the other predefined eHealth characteristics were associated with adherence. This could suggest that the eHealth device itself is not the primary driver of nonadherence. However, our findings must be interpreted with caution, as the analysis lacks statistical power due to the small sample size of included studies. Additionally, the heterogeneity of the included devices further limits the robustness of our conclusions. This needs further clarification, as our results indicated that mental overload was reported in only one of the included studies as a factor influencing patient adherence. Previous studies have highlighted the ease of use of technology as a crucial facilitator for eHealth implementation [90]. Thus, patient adherence may depend less on the workload and more on the device’s accessibility [91], and future devices should focus on an appropriate front-end design to optimize patient engagement. Clearly, these findings must be interpreted with caution, given the limited evidence available.

In addition to patients’ ability to use eHealth devices, it is essential that health care professionals also trust these technologies. A major concern among health care providers regarding the implementation of eHealth is the apprehension that older patients or those with more severe diseases may not be able to use the technology effectively [92]. However, this perception is not corroborated by our findings, as most studies that reported on these parameters did not find a negative association between adherence and either age or NYHA classification. In fact, adherence even improved with increasing age in a substantial number of studies. Health care professionals play a crucial role in providing the necessary clinical information to tailor the device to the patient’s needs and should be actively involved in the implementation of eHealth devices in HF management. Additionally, these findings might also encourage health care policy makers to extend eHealth implementation to broader patient populations, rather than limiting it to specific patient characteristics, while emphasizing the need to improve long-term commitment to these devices.

Effective patient engagement is essential for the successful implementation of eHealth solutions in HF management and alleviating the HF burden on health care systems. Yet, clinical studies exploring the impact of eHealth frequently provide minimal information on patient engagement, underscoring a gap in current research methodologies. In addition, the identification of factors influencing adherence is crucial for successful implementation. This should include patients who refuse to use eHealth devices. Future studies should provide a more comprehensive description of patient adherence, including information on the frequency and intensity of eHealth use, as described in the CONSORT-EHEALTH (Consolidated Standards of Reporting Trials of Electronic and Mobile Health Applications and Online Telehealth) checklist [93], or the World Health Organization mHealth evidence reporting and assessment checklist [94]. This will ensure better comparability across studies and enhance understanding of adherence behaviors. Furthermore, more research has to be conducted to study suited strategies for enhancing patient engagement. For example, financial incentives have been shown to improve patient adherence to eHealth devices in HF [45]. A similar effect is hypothesized for gamification, the use of game design elements in nongaming settings, although evidence supporting this remains limited [46]. To advance research in this field, HF specialists should be more actively involved in the development and implementation of eHealth solutions. This requires greater interdisciplinary collaboration, including engagement with patients to assess their needs and with software developers to ensure that eHealth tools align with both patient and clinical requirements. Additionally, educating health care professionals about eHealth possibilities can be highly valuable in fostering adoption and effective use.

### Limitations

We exclusively used quantitative data to characterize adherence behaviors. While qualitative data from interviews or focus groups could offer deeper insights, our objective was to present trends in adherence as objectively as possible. To enable a meaningful aggregation of the results and quantify the relationships between adherence and eHealth characteristics, outcome measures needed to be comparable. Therefore, we had to exclude nearly 50% (n=35) of the included studies from the inferential statistical analysis, resulting in a small sample size. Due to this small sample size, our study might lack sufficient power to detect a relationship between adherence and eHealth characteristics, thereby increasing the risk of type II errors. The inclusion of studies was restricted to English-language publications to ensure consistency in reporting and interpretation. However, this may have introduced language bias, potentially leading to the exclusion of relevant findings from non-English sources. Nonetheless, as most high-impact papers are published in English, we are confident that the risk of missing substantial data is minimal. Comparing adherence across different device types could have provided valuable guidance for clinical practice. However, this analysis was not feasible due to inconsistencies in adherence reporting, varying adherence definitions across studies, and the small sample sizes within each device category.

### Comparison With Prior Work

Recent reviews on patient adherence have primarily focused on chronic diseases broadly, with HF often being underrepresented [12]. Furthermore, a recent review by Madujibeya et al [11] specifically addressing adherence to eHealth in HF only included mHealth while neglecting other eHealth categories. Consequently, we offer a more targeted and comprehensive review of adherence data concerning eHealth in HF. Still, their results are comparable to ours, as they also found a lack of consistency in reporting patient adherence and observed trends toward decreased use of eHealth over time.

### Conclusions

Reporting and definition of patient adherence in HF studies lack completeness and consistency. Overall, trends toward a decrease of eHealth use over time could be identified, and tailoring the devices to meet the patients’ needs might overcome this problem. Future studies should provide a more comprehensive description and definition of adherence to identify factors improving patient compliance with eHealth.

## Supplementary material

10.2196/63409 Multimedia Appendix 1 Searching strategy.

10.2196/63409 Multimedia Appendix 2 Overview of included studies.

10.2196/63409 Checklist 1 PRISMA-ScR (Preferred Reporting Items for Systematic Reviews and Meta-Analyses Extension for Scoping Reviews) checklist.

## Abbreviations

CONSORT-EHEALTH: Consolidated Standards of Reporting Trials of Electronic and Mobile Health Applications and Online Telehealth
HF: heart failure
MeSH: Medical Subject Headings
mHealth: mobile health
NYHA: New York Heart Association
PRISMA-ScR: Preferred Reporting Items for Systematic Reviews and Meta-Analyses extension for Scoping Reviews
