# Supplementary material for: Adherence to eHealth Interventions Among Patients With Heart Failure: Scoping Review
Source: JMIR Mhealth Uhealth. 2025 Jun 27;13:e63409. doi: 10.2196/63409 (PMC12227154; doi:10.2196/63409)
Supplement: Multimedia Appendix 1 [file mhealth-v13-e63409-s001.docx]

Searching strategy Pubmed

("Patient Compliance"[Mesh] OR "Patient Participation"[Mesh] OR adheren*[tiab] OR complian*[tiab] OR persisten*[tiab] OR cooperat*[tiab] OR engag*[tiab]) AND ("Telemedicine"[Mesh]OR eHealth OR mHealth OR telemedic*[tiab] OR telemonitor*[tiab] OR mobile health) AND ("Heart Failure"[Mesh] OR heart fail*[tiab] OR cardiac fail*[tiab] OR myocardial fail*[tiab] OR diastolic fail*[tiab] OR systolic fail*[tiab] OR heart dysfunction*[tiab] OR diastolic dysfunction*[tiab] OR systolic dysfunction*[tiab] OR heart decompensation*[tiab])

Searching strategy CINAHL

(Patient compliance or patient adherence OR patient participation OR patient cooperation OR patient engagement) AND (telemedicine or telehealth or ehealth or e-health or mhealth or m-health) AND (heart failure or cardiac failure or chf or chronic heart failure or congestive heart failure)

Searching strategy PsycInfo

(Patient compliance or patient adherence OR patient participation OR patient cooperation OR patient engagement) AND (telemedicine or telehealth or ehealth or e-health or mhealth or m-health) AND (heart failure or cardiac failure or chf or chronic heart failure or congestive heart failure)
