# Supplementary material for: Adherence to eHealth Interventions Among Patients With Heart Failure: Scoping Review
Source: JMIR Mhealth Uhealth. 2025 Jun 27;13:e63409. doi: 10.2196/63409 (PMC12227154; doi:10.2196/63409)
Supplement: Multimedia Appendix 2 [file mhealth-v13-e63409-s002.docx]

| **First author** | **Year of publication** | **Description of intervention** | **Parameters collected by intervention (frequency)** | **Adherence** |
| --- | --- | --- | --- | --- |
|  |  |  |  |  |
| Apergi et al* [25] | 2021 | Amazon’s Alexa+ (voice-activated survey) or Avatar via tablet (visually animated and voice-enabled) | Symptoms, treatment compliance (all daily) | Alexa+: mean usage of 35.3 times, Avatar: mean usage of 37.8 times |
| Athilingam et al* [47] | 2017 | HeartMapp app with biometric sensor (Zephyr BioHarness-3 chest strap) | Weight, symptoms, physical activity, medication adherence, heart rate (daily) | Mean adherence: 78% (days application was used/study days) |
| Auton et al* [48] | 2023 | Luscii app with body mass scale, blood pressure monitor, pulse rate monitor | Body mass, blood pressure, heart rate (at T0 and T3) | Adherence heart rate: 100% (% of patients submitting data at both time points); adherence blood pressure: 96%, body mass: 89% |
| Barbaric et al* [23] | 2022 | Medly voice app | Weight, symptoms, blood pressure, heart rate (daily) | Adherence: 73% (overall adherence) |
| Blockhaus et al [49] | 2022 | LifeVest (wearable cardioverter-defibrillator) | Physical activity, body position, heart rate (as often as possible) | Adherence: 21.39 hours (mean hours life west was worn per day) |
| Blomqvist et al* [50] | 2024 | Activity coach computer program with accelerometer (ActiGraph GT9X) | Physical activity (daily) | Median adherence: 61% (days of activity was documented/study days) |
| Blomqvist et al* [51] | 2025 | Activity coach app with accelerometer (ActiGraph GT9X) | Physical activity (daily) | Adherence: 69% (mean days the activity coach app was used) |
| Boyne et al* [52] | 2014 | Health Buddy device (liquid crystal display and four keys connected to a landline phone) | Symptoms, signs (daily) | Overall adherence: 90% (days with response/study days) |
| Brons et al [20] | 2023 | e-Vita HF online platform, weight scale, blood pressure monitor | Weight, blood pressure, heart rate (daily) | Adherence weight: 74% (% of patients with 3 or more times a week for at least 42 week within 1 year); Adherence weight, blood pressure and heart rate (combined): (67%) |
| Carter et al* [53] | 2023 | App with biometric sensor, digital weight scale and a digital blood pressure monitor | Weight, symptoms, signs, physical activity, heart rate, blood pressure, oxygenation (daily) | Adherence sensor: 82.2% (days sensor was warn/study days), average of 13.5 h/d. Adherence blood pressure monitor: average of 1.2 times per day; Adherence digital weight scale: average of 1.1 times per day; Adherence symptom questionnaire: 71% (Days questionnaire was completed/study days) |
| Chausiaux et al [54] | 2024 | Weight scale, AI-enabled peripheral edema monitor | Weight, edema | Adherence: 71.3% (% of patients with 16 or more days per month using the AI monitor), 5.7% (weight scale) |
| Clark et al [43] | 2007 | Telephone support | Weight, symptoms, signs (at least once a month) | Adherence: 65.8% (% of patients submitting data once a month or more) |
| de Lusignan et al* [44] | 2001 | Video consultation equipment with NAIS memory Blood Pressure Watch and weight scale | Weight, symptoms, blood pressure, heart rate (vital signs daily; symptoms weekly for 3 month, than biweekly for 3 month, then monthly) | Weight: Mean adherence: 74% (days data was transmitted/study days); Blood pressure and pulse: Mean adherence: 90% |
| Ding et al [32] | 2020 | Weight scale | Weight (daily) | Adherence weight: 74% (% of patients with 4 or more days with data transmission a week) |
| Dorsch et al [55] | 2021 | Manage  HF4Life app with FitBit physical activity monitor and FitBit weight scale | Weight, symptoms, physical activity | Median adherence: 63 days (number of days the app was used for self-monitoring) |
| Eilat-Tsanani et al* [56] | 2016 | Tele-Weight weight scale | Weight (daily) | Mean adherence: 56% (% of days with data transmission) |
| Evans et al [57] | 2016 | Touchscreen tablet, watch with built-in accelerometers, blood pressure monitor, weight scale | Weight, symptoms, physical activity, blood pressure, heart rate, skin temperature (daily) | Adherence watch: 73% (patients wear watch 24/7). Adherence daily blood pressure monitoring: 72% (of patients). Adherence daily weight monitoring: 77% (of patients). Adherence daily survey taken: 64% (of patients) |
| Galinier et al [7] | 2020 | Telehealth device, weight scale | Weight, symptoms (daily) | Median adherence body weight: 74.6% (% of patients with daily recording) |
| Gardner et al* [58] | 2016 | iPad with weight scale, blood pressure monitor, pulse oximeter, actigraph | Weight, symptoms, signs, physical activity, blood pressure, heart rate, oxygenation (daily) | Mean adherence (mean days used): Weight scale: 5, blood pressure monitor: 6, ipad: 6, pulse oximeter: 6, acitgraph: 6 |
| Guo et al [59] | 2019 | Telehealth platform (via app or website), weight scale, blood pressure monitor, wearable ECG monitor or ECG recorded by app, oxygen saturation monitor | Weight, symptoms, blood pressure, heart rate, heart rhythm, oxygenation (weekly) | Adherence: 61% (% of patients using the platform more than once a week) |
| Guzman-Clark et al* [39] | 2013 | Health Buddy device (liquid crystal display and four keys connected to a landline phone) | Symptoms, signs (daily) | Mean adherence: 54.9 days (days with response/study days) |
| Guzman-Clark et al* [27] | 2021 | Home Telehealth Program with wearables | N/A | Mean adherence: 57.1% (% of days patients logged in) |
| Haynes et al* [33] | 2020 | Telehealth device, weight scale, blood pressure monitor, heart rate monitor | Weight, symptoms, blood pressure, heart rate (daily) | Adherence weight: 53.3% (% of study days with weight transmission) |
| Haynes et al * [34] | 2020 | CardioMEMS device, CardioMEMS pillow | Pulmonary artery pressure (daily) | Adherence: 77.6% |
| Haynes et al* [28] | 2021 | Monitor, weight scale, blood pressure monitor, heart rate monitor | Weight, symptoms, blood pressure (daily) | Adherence: 49% (% of days weight was transmitted) |
| Ho et al [60] | 2021 | Touchscreen tablet computer, blood pressure monitor, pulse oximeter, weight scale | Weight, symptoms, heart rate, blood pressure, oxygenation (daily) | Adherence: 94% (% of patients) |
| Hovland-Tånneryd et al [35] | 2019 | OPTILOGG device: specialised tablet computer with app and weight scale | Weight, symptoms (weight daily, symptoms every 5 days) | Median adherence: 94% (% of days patients used the eHealth tool) |
| Johnston et al* [61] | 2022 | App, FitBit activity tracker, FitBit weight scale | Weight, symptoms, physical activity, sleep time, heart rate (daily) | Activity tracker: 78% of participants had 100% adherence (days tracker was worn/study days), 22% of participants had 93% adherence; weight scale: 56% of participants had 100% adherence (days weight was submitted/study days) |
| Kastner et al* [16] | 2010 | App with blood pressure monitor and body weight scale | Weight, symptoms, medication adherence, blood pressure, heart rate (daily) | Adherence: 78% (days a complete set of values was sent) |
| Kagiyama et al [19] | 2024 | Tablet (data transmission), weight scale, sphygmomanometer,thermometer, pulse oximeter, handheld sound recorder | Weight, blood pressure, heart rate, temperature, saturation, ECG, PCG | Adherence: 75% (% of patients with at least 70% of days with self-measurement) |
| Keogh et al* [62] | 2024 | App, FitBit activity tracker, Aria Air smart scale | Weight, symptoms, physical activity, heart rate, sleep (vitals daily, symptoms 1xmonth or if vitals changed) | Adherence tracker: 86.2% (mean days of usage); adherence weight measurements: 73.7% |
| Kitsiou et al [63] | 2025 | iCardia4HF app, weight scale, blood pressure monitor, FitBit activity tracker | Weight, symptoms, blood pressure, heart rate, physical activity (daily) | Adherence: 86.4% (weight), 86.2% (blood pressure) |
| Koehler et al [15] | 2011 | App, blood pressure monitor, body weight scale, 3-lead ECG | Weight, symptoms, blood pressure, heart rate | Adherence: 81% (% of patients with at least 70% of daily data transfers and no break for >30 days (except during hospitalizations)) |
| Ledwidge et al* [64] | 2013 | HeartPhone app, weight scale | Weight (daily) | Adherence: 90.6% (percentage of days with data transmission) |
| Lieback et al* [65] | 2012 | Cardio Messenger (Bedside transmitter of ICD data), weight scale, blood pressure monitor | Weight, blood pressure, heart rate, heart rhythm, patient activity (daily) | Adherence ICD: 82% (% of all days when transmission was expected), adherence weight: 78%, adherence blood pressure: 76% |
| Lloyd et al* [66] | 2019 | Penn State Heart Assistant: Web-based, tablet computer-accessed telehealth platform | Weight, exercise, medication adherence (all daily) | Adherence: 84% (% of data that was submitted) |
| Marier-Tétrault et al [67] | 2024 | App, weight scale, blood pressure monitor, glucometer | Weight, symptoms, blood pressure, heart rate, glucose (daily) | Adherence: 47% (% of patients with >80% of days using the app) |
| Mohapatra et al [45] | 2024 | App, FitBit activity tracker, weight scale | Weight, symptoms, physical activity (daily) | Adherence: 50-70% (weight), 60-75% (activity tracker), 56-65% (symptoms) |
| Morak et al* [40] | 2011 | App, blood pressure monitor, single channel ECG recorder, body weight scale | Weight, symptoms, blood pressure, heart rate, heart rhythm, medication adherence (twice a day) | Adherence: 82.2% (at an expected rate of 13 data sets per patient) |
| Morgan et al [68] | 2017 | Patients with cardiac implanted electronic devices (CIED) downloaded data from that device | Depending on device (weekly) | Adherence: 58% (% of patients transmitted data for at least 75% of study weeks, not including any periods of hospitalization, after 6 month) |
| Mortara et al* [41] | 2009 | Telephone support, blood pressure monitor, weight scale, holter-style recorder | Weight, symptoms, blood pressure, heart rate, cardiorespiratory activity, physical activity (symptoms once a month, rest daily) | Adherence vital signs: 81% (% of requested data that was transmitted); adherence cardiorespiratory recordings: 92% |
| Murugappan et al* [69] | 2024 | Heartfelt device, weight scale | Weight, foot volume (daily) | Adherence Heartfield: 76% (mean days of usage); adherence weight: 10% |
| Nederend et al [70] | 2021 | HealthMate app with blood pressure monitor, weight scale, step counter watch, rhythm monitor (single-lead ECG) | Weight, physical activity, blood pressure, heart rate, heart rhythm (weight and blood pressure twice a week, physical activity daily, heart rhythm in case of palpitations) | Adherence weight and blood pressure monitoring: 50% (patients with weekly measurements ). Adherence step counter: 50% (of patients wearing step counter daily) |
| Nouryan et al [71] | 2019 | Telehealth station with video monitor, blood pressure monitor, stethoscope, weight scale, pulse oximetry monitor | Weight, symptoms, signs, blood pressure, heart rate, oxygenation | Adherence: 64% (patients with at least 20 uploads during follow-up) |
| Petrie et al [72] | 2024 | App, blood pressure monitor, weight scale | Weight, symptoms, blood pressure, heart rate (daily) | Adherence: 52% (all vitals reported every day) |
| Piette et al [42] | 2008 | Touch-tone telephone support | Weight, symptoms, medication availability, medication adherence (weekly) | Adherence: 92% (% of successful assessment attempts) |
| Ploux et al [26] | 2021 | CareLine Solutions™ app with body weight scale and a blood pressure monitor | Weight, symptoms, blood pressure, heart rate | Adherence: 84% (1 month before lockdown), 87% (first month during lockdown) |
| Prescher et al* [21] | 2023 | Tablet, weight scale, ECG device, blood pressure monitor | Weight, symptoms, blood pressure, heart rate (daily) | Adherence all 4 vital parameters: 89.1% (days all parameters collected / days in study); adherence blood pressure monitoring: 93.71%; adherence weight: 92.4%; adherence ECG: 92.1% |
| Prescher et al [38] | 2014 | App, blood pressure monitor, body weight scale, 3-lead ECG | Weight, symptoms, blood pressure, heart rate | Adherence: 88.9% (% of patients transmitting at least 1 vital parameter a day) |
| Radhakrishnan et al* [30] | 2021 | Heart Health Mountain app with sensor-controlled digital gaming | Weight, physical activity (daily) | Adherence serious gaming: 71% (patients using gaming for more than 50% of study days). Adherence monitoring: 80% (patients transmitted weight data for 5 or more days a week) |
| Rao et al [73] | 2022 | HealthStar app | N/A | Adherence: 67% (% of patients using the app after 4 weeks) |
| Rosen et al [36] | 2017 | Telehealth platform (assessed via touch screen tablet), weight scale | Weight, symptoms, medication adherence | Median adherence: 96% (% of days, data was transmitted) |
| Sabatier et al* [24] | 2022 | Telehealth platform | Weight, symptoms, physical activity, blood pressure, heart rate, lifestyle/diet, fatigue, morale, treatment compliance (weight, heart rate, blood pressure, symptoms = 3 times a week) (lifestyle = 2 times a week) (fatigue, morale, treatment compliance = 1 a week) | Adherence: 65.7% (mean % of anticipated monitoring sessions) |
| Schacksen et al [74] | 2021 | HeartPortal webpage | Weight, symptoms, physical activity, blood pressure, heart rate, respiration, hours of sleep (symptoms twice a month, frequency of other parameters unclear, all data self-reported) | Adherence: 74.93% (% of questionnaires completed) |
| Scherrenberg et al* [75] | 2023 | HF coach app and digital health platform, blood pressure monitor, weight scale, wearable chest patch | Weight, symptoms, physical activity, blood pressure, heart rate, body posture (daily) | Adherence blood pressure: 96.2% (of study days); adherence weight: 93.6%; adherence symptoms registration: 69.3% |
| Schmaderer et al [76] | 2023 | Play-It Health App | Weight, medication adherence | Adherence weight: 61% (% of patient recording 50% of time or more, mHealth); 25% (mHealth+) |
| Seto et al [84] | 2012 | App with weight scale, blood pressure monitor, ECG recorder | Weight, symptoms, blood pressure, heart rate, heart rhythm (Heart rhythm weekly, rest daily) | Adherence: 70% (% of patients completed at least 80% of their possible daily readings) |
| Siebermair et al [37] | 2015 | CareLink™ system, handheld telemetry wand (for data transmission of ICDs, CRT, pacemakers) | Arrhythmia occurrence (once) | Adherence: 76.1% (% of patients completing initial data transmission) |
| Smeets et al* [77] | 2018 | CardioCoach app with weight scale and blood pressure monitor | Weight, symptoms, signs, medication adherence, heart rate, blood pressure, self-care | Adherence vital sign registration: 94.6% |
| Sohn et al* [31] | 2020 | FitBit activity tracker with BodyTrace scale and smart pill bottles | Weight, physical activity, medication adherence, heart rate, sleep duration, capillary oxygenation (daily) | Median adherence activity tracker: 79.1% (hours activity tracker was worn). Adherence bathroom scale: 59.7% (days scale was used). Adherence smart pill bottle: 2.8% (days bottle was used). Usage of applications decreased over time (Activity tracker with 5.4%, weight scale with 20.3%, bottle with 9.7% |
| Triantafyllidis et al [78] | 2015 | Tablet computer with blood pressure monitor, weighing scale, pulse oximeter | Weight, symptoms, blood pressure, heart rate, oxygenation (frequency N/A) | Mean adherence: 4.96 (number of days in a week, vital parameters and/or symptoms were reported) |
| Vuorinen et al* [79] | 2014 | App, weight scale, blood pressure monitor | Weight, symptoms, blood pressure, heart rate (weekly) | Adherence weight: 86% (proportion of weekly submitted self-measurements); adherence blood pressure, heart rate, symptoms reporting: 89% |
| Wakefield et al [80] | 2009 | EHC 200 Sentinel Patient Station (videophone) or telephone contact with HF nurse | Medication adherence, knowledge, self-efficacy, satisfaction with care (frequency not reported) | Telephone group: Adherence 94% (% of completed scheduled contacts); Videophone group: Adherence 96% |
| Ware et al* [17] | 2019 | Medly smartphone app, weight scale, blood pressure monitor | Weight, symptoms, blood pressure, heart rate (daily) | Adherence: 73.6% (% of days patients took all 4 morning readings |
| Wei et al* [81] | 2021 | Habit Heart App with weight scale | Weight, symptoms (daily) | Adherence: 50% (≥1 interaction with the app per day/study days) |
| Yokota et al [82] | 2023 | Mimamori-cho app with blood pressure monitor, body composition monitor, digital thermometer, pulse oximeter, pedometer with an accelerometer | Weight, symptoms, physical activity, blood pressure, heart rate, body temperature, body fat, oxygen saturation, sleep quality, self-care (daily) | Median adherence: Blood pressure, body weight, body temperature: 100% (days data was submitted/study days); Median adherence: Oxygen saturation: 100% |
| Yoon et al* [18] | 2024 | App, weight scale, blood pressure monitor, bioimpedance device | Weight, symptoms, blood pressure, heart rate, body water (daily) | Adherence: 80% (mean logins app), 58.6% (mean days symptoms entered) |
| Zan et al [83] | 2015 | iGetBetter online platform (assessed with iPad Mini tablet computer) with interactive voice response, weight scale, blood pressure monitor | Weight, symptoms, blood pressure, heart rate | Adherence: ≥80% (of daily usage in >50% of participants) |
| Ziacchi et al* [22] | 2023 | MyTriage app | Symptoms, signs, medication adherence (daily) | Mean adherence: 49.3% |
| Zisis et al [29] | 2021 | App | Weight (daily) | Adherence: 20% of participants completed ≥70% of the full program, 80% did not engage at all |

Studies marked with “*” were included in the inferential statistical analysis; ECG=electrocardiogram; ICD=implantable cardioverter defibrillator; mHealth=mobile health; N/A=not available; PCG=phonocardiogram.
